# Comprehensive Neuropsychiatric and Cognitive Characterization of Former Professional Football Players: Implications for Neurorehabilitation

**DOI:** 10.3389/fneur.2019.00712

**Published:** 2019-08-07

**Authors:** Alex R. Terpstra, Brandon P. Vasquez, Brenda Colella, Maria Carmela Tartaglia, Charles H. Tator, David Mikulis, Karen D. Davis, Richard Wennberg, Robin E. A. Green

**Affiliations:** ^1^Cognitive Neurorehabilitation Sciences Laboratory, Toronto Rehabilitation Institute, Toronto, ON, Canada; ^2^Department of Psychology, University of British Columbia, Vancouver, BC, Canada; ^3^Neuropsychology & Cognitive Health, Baycrest, Toronto, ON, Canada; ^4^Canadian Concussion Centre, Toronto Western Hospital, Toronto, ON, Canada; ^5^Division of Neurology, Krembil Neuroscience Centre, University Health Network, University of Toronto, Toronto, ON, Canada; ^6^Tanz Centre for Research in Neurodegenerative Disease, University of Toronto, Toronto, ON, Canada; ^7^Division of Neurosurgery, Krembil Neuroscience Centre, Toronto Western Hospital, University of Toronto, Toronto, ON, Canada; ^8^Division of Neuroradiology, Joint Department of Medical Imaging, Toronto Western Hospital, University of Toronto, Toronto, ON, Canada; ^9^Division of Brain, Imaging and Behaviour – Systems Neuroscience, Krembil Research Institute, University Health Network, Toronto, ON, Canada; ^10^Department of Surgery, University of Toronto, Toronto, ON, Canada; ^11^Institute of Medical Science, University of Toronto, Toronto, ON, Canada; ^12^Department of Psychiatry, Faculty of Medicine, University of Toronto, Toronto, ON, Canada

**Keywords:** sports concussion, neuropsychiatric functioning, cognitive dysfunction, executive function, neurorehabilitation

## Abstract

**Objectives:** To identify novel targets for neurorehabilitation of people with a remote history of multiple concussions by: (1) comprehensively characterizing neuropsychiatric and cognitive functioning in former professional football players, with a focus on executive functions; (2) distinguishing concussion-related findings from pre-morbid/cohort characteristics of professional football players; and, (3) exploring the relationship between executive functions and neuropsychiatric symptoms.

**Participants:** Sixty-one high-functioning former professional football players and 31 age- and sex-matched control participants without history of concussion or participation in contact sports.

**Design:** Between-groups analyses.

**Main measures:** *Neuropsychiatric*. Personality Assessment Inventory (PAI) clinical scales plus the Aggression treatment consideration scale; the Mini International Neuropsychiatric Interview (MINI). *Cognitive*. Comprehensive clinical neuropsychological battery assessing domains of verbal and visuospatial attention; speed of processing and memory; current and estimated pre-morbid IQ; and, executive functioning, including two experimental measures that were novel for this population (i.e., response inhibition and inconsistency of responding on a go/no-go task).

**Results:** (1) Compared to control participants, former professional football players scored significantly higher on the PAI Depression, Mania, and Aggression scales, and significantly lower on response inhibition. (2) Relative to controls, former players with >3 concussions ($\bar{\text{x}}=6.1$), but not former players with ≤ 3 concussions ($\bar{\text{x}}=2.0$), showed (i) significantly higher scores on the PAI Depression scale, (ii) significantly more MINI clinical diagnoses overall, and manic/hypomanic episodes specifically, and (iii) significantly poorer executive function. (3) Mediation analysis revealed that concussion exposure had a significant indirect effect on PAI Depression, Mania, and Aggression via inconsistency of responding on the go/no-go task.

**Conclusions:** Notable impairments to neuropsychiatric functioning and worse performance on a sensitive experimental measure of executive function were observed; these were related to both concussion history and pre-morbid (cohort) factors. Therefore, neuropsychiatric and executive functioning should be carefully assessed in those with a remote history of multiple concussions. Moreover, former players' neuropsychiatric symptoms were associated with inconsistency of responding; this suggests that treatments targeted at response inconsistency could help to mitigate neuropsychiatric dysfunction.

## Introduction

Repetitive concussions are a growing public health concern due to their cumulative effects and evidence of down-stream neurodegenerative consequences (1–6). American football players endure higher concussion exposure (7) and thus former players can provide a window into the delayed effects of multiple concussions in the context of contact sport. While post-mortem research has established a link with chronic traumatic encephalopathy (CTE) in this population (3, 5, 6), there remains a gap in our understanding of the long-term neuropsychiatric and cognitive sequelae in retired players measured *in vivo*.

Studies examining *neuropsychiatric* function in former professional football players have focused predominantly on depression, with significantly higher symptom endorsement and rates of clinically elevated depression relative to control participants (8–14). In post-mortem studies using proxy reports of retired players who had developed CTE, symptoms of “explosivity,” “impulsivity,” “aggression,” and “paranoia” have been reported (6, 15). In a pilot study of 17 former professional football players without a diagnosis of dementia, we observed significantly higher mania symptoms and aggression on the Personality Assessment Inventory (PAI) (16) as compared to control participants. In the former players, higher aggression scores were negatively associated with orbitofrontal cortex thickness and uncinate fasciculus axial diffusivity (17). Few other studies to date, however, have examined neuropsychiatric symptoms other than depression *in vivo*.

In studies examining *cognitive* functioning in former professional football players, learning and memory impairments have been the predominant deficits studied and observed (8, 14, 18–22). There is also some evidence that executive functioning is affected in retired professional football players (23) and related populations (e.g., former high school and college athletes with a history of multiple concussions) (24, 25). Executive functions refer to higher order mental control processes responsible for the management of behavior; they allow us to adapt to our environment from moment to moment as a function of current goals. One core component of executive functioning is inhibitory control, which can be generally defined as the ability to override an automatic behavior to achieve a task specific objective (26, 27).

A small number of studies have examined traditional measures of executive functioning in retired football players (e.g., verbal fluency, Trail Making Test B) (14, 22, 28), and impairments were found in one study (22). In our pilot study of retired professional football players mentioned above, performance on an experimental measure of inhibitory control (commission errors on a go/no-go task) was lower in retired players relative to control participants. More commission errors on this task were also negatively associated with orbitofrontal cortex thickness and uncinate fasciculus axial diffusivity in the former players (17).

An experimental measure of executive functioning that has yet to be used with this population is intra-individual variability (IIV) of reaction time, which represents inconsistency of responding across trials of a task. IIV has shown notable sensitivity to executive impairments in brain injury (29, 30). One study found ongoing impairments in IIV in otherwise fully cognitively recovered TBI patients (29). A study by our group found declines in IIV during the chronic stages of moderate–severe TBI, from 1 to 2+ years post-injury (30).

Disruption to executive functioning has been implicated in a predisposition for and maintenance of neuropsychiatric illnesses, including depression and mania (31–33). Given that higher concussion exposure may contribute to poorer executive functioning (24, 25), these findings raise the question of whether concussion-related executive functioning impairments might increase susceptibility to neuropsychiatric symptoms. To date, no previous study has examined whether executive and neuropsychiatric functioning are directly related in this population, nor whether executive function variables might mediate (i.e., give rise to) the relationship between concussion exposure and neuropsychiatric symptoms. If so, remediating executive dysfunction could prevent or mitigate expression of neuropsychiatric dysfunction in people with a history of multiple concussions.

The overarching aim of this study was to identify novel treatment targets for the long-term cognitive and neuropsychiatric sequelae of multiple concussions. Our first objective was to replicate and extend previous studies characterizing neuropsychiatric and cognitive functioning of former professional football players with a remote history of multiple concussions. Here, we comprehensively measured neuropsychiatric and cognitive functioning in retired players with a history of multiple concussions, and we added novel experimental measures of executive function. These were IIV and response inhibition on a go/no-go reaction time task. Based on past research (12, 17), we predicted greater symptoms of depression, mania, and aggression in comparison to age- and education-matched control participants. Also based on previous findings (8, 17, 19, 22), we predicted worse performance relative to control participants on tests of learning, memory, and executive functioning.

Our second objective was to discern whether any observed differences between retired players and control participants on our neuropsychiatric and cognitive measures were attributable to concussion history or, rather, to pre-morbid, cohort characteristics. To do this, we examined whether higher vs. lower concussion exposure—following the precedent of Guskiewicz et al. (12)—was associated with neuropsychiatric and cognitive outcomes. Greater impairments in the higher concussion exposure group for measures of depression, mania, and aggression, and for measures of learning, memory, and executive functioning, which we predicted based on past research (34–38), would offer evidence that impairments are secondary to concussion history rather than to pre-morbid traits.

Finally, we explored the *relationship* between cognitive and neuropsychiatric functioning in former professional football players as a function of concussion exposure. Our pilot research showed evidence that executive functioning deficits and neuropsychiatric elevations correlated with common structural findings (17); as well, studies of other populations have shown a relationship between executive functioning deficits and neuropsychiatric illness (31–33). Thus, we anticipated a relationship between executive functioning measures and neuropsychiatric findings in the former players. We also anticipated a possible mediating role of executive functioning in the relationship between concussion exposure and neuropsychiatric symptoms. In other words, in the absence of executive function impairments, symptoms might be less likely to manifest in people with a remote history of multiple concussions.

## Methods

### Participants

Sixty-one former male Canadian Football League (CFL) players were recruited from across Canada through advertisements in alumni newsletters, presentations, and word of mouth by the Canadian Concussion Centre. Primary inclusion criteria: history of play in the CFL for 3 years or more, under 85 years of age, and currently employed or retired for reasons unrelated to disability (all participants were gainfully employed or voluntarily retired). Primary exclusion criteria: diagnosis of dementia, history of stroke, systemic illness (e.g., diabetes, lupus).

Former players were divided into higher and lower concussion exposure subgroups following the precedent of Guskiewicz et al. (12). Note that for 17 of the 61 retired athletes, partial data has been previously reported (17, 39).

Thirty-one male control participants ranging in age from 28 to 70 were recruited from the local community with the same exclusions as above. Additional exclusions were history of concussion, participation in contact sport (to reduce risk of undocumented concussions), and 2 z-scores below average or more on any neuropsychological test (40). Control participants were closely group-matched to the retired players on age, education, and estimated pre-morbid IQ (Table 1).

**Table 1:** Demographics and concussion history of former professional football players, concussion exposure subgroups, and control participants.

**Demographic and injury variables**	**All retired athletes (*N* = 61)**	**Lower concussion exposure (≤3; *n* = 26)**	**Higher concussion exposure (≥4; *n* = 35)**	**Control participants (*N* = 31)**
**M (SD) Range**				
Age	55.0 (12.8)	55.7 (14.4)	54.5 (11.8)	49.7 (12.1)
	28–84	28–84	31–82	28–70
YOE	15.9 (1.8)	15.6 (1.8)	16.1 (1.7)	16.5 (2.3)
	12–21	12–20	12–21	13–22
Estimated premorbid IQ^†^	112.5 (7.8)	112.5 (7.0)	112.5 (8.6)	112.3 (8.8)
	95–125	95–122	95–125	
No. of years played in CFL	7.5 (3.6)	8.3 (3.7)	6.7 (3.5)	
	1–15	1–14	3–15	
No. of self-reported concussions	4.4 (2.8)	2.0 (0.9)	6.1 (2.4)	
	0–13	0–3	4–13	
Age of first concussion	19.7 (6.0)	22.1 (7.2)	18.4 (4.9)	
	8–33	8–33	9–32	
Age of last concussion	24.7 (4.5)	23.8 (5.0)	25.2 (4.3)	
	17–34	17–33	20–34	
Years since last concussion	30.7 (14.6)	34.9 (17.2)	28.6 (13.0)	
	5–58	5–58	5–49	
Years since last play	23.7 (13.8)	24.2 (14.3)	23.4 (13.6)	
	0–53	0–53	1–53	

*M, mean; SD, standard deviation; YOE, years of education*.

† *Estimated pre-morbid IQ was collected with the Wechsler Test of Adult Reading (WTAR) during neuropsychological assessment*.

### Measures

All participants were administered a neuropsychiatric assessment comprising the PAI (16) and the Mini International Neuropsychiatric Interview (41) (MINI), which are widely used in TBI and have strong psychometric properties (42, 43). The PAI is a self-report instrument of personality and neuropsychiatric function, with validity demonstrated by our group for TBI for all scales except the *Somatization* and *Schizophrenia* scales, which were excluded from this study (43). We report here on all remaining clinical scales, plus the *Aggression* treatment consideration scale. The MINI is a widely employed, structured, diagnostic interview that measures neuropsychiatric function and screens for 15 disorders based on DSM-IV criteria. We present here findings for all major Axis I diagnoses.

The cognitive battery included traditional clinical neuropsychological tests selected for known validity and reliability for TBI (see Supplementary Table 1 for test name, description, and outcome measure). Experimental measurement of executive functioning was undertaken using the Sustained Attention to Response Task (SART), a go/no-go reaction time test. The SART was designed as a measure of sustained attention (44), a capacity that is developmentally linked to inhibitory control (45); and, as a go/no-go test, it has been employed to examine inhibitory control (34). Two SART outcomes were employed. The first was commission errors on the task (measuring inhibitory control). The second was IIV of reaction time (measuring inconsistency of responding). For IIV, standard deviation of the correct reaction time was recorded, from which a coefficient of variation was calculated as an index of IIV (i.e., SD/mean RT) (46, 47).

### Design and Procedures

A between-subjects design was employed. All participants provided their informed consent and were tested face-to-face with all measures completed in one testing session. All neuropsychiatric and neuropsychological tests were administered by a trained psychometrist or post-doctoral trainee. Inter-rater reliability was established between testers. The study was approved by the University Health Network Research Ethics Board, Toronto, ON, Canada.

### Analysis

Objective 1: Analyses were conducted using the Statistical Package for the Social Sciences, version 21 (48). To compare former players to control participants on the demographic, neuropsychiatric, and cognitive measures, we employed between-group, independent *t*-tests. We used an alpha level of 0.05 (1-tailed) for directional hypotheses, and 0.05 (2-tailed) plus Holm-Bonferroni adjustment for multiple comparisons (49) for exploratory comparisons.

To examine the frequency of neuropsychiatric dysfunction in the groups, we compared the number of individual cases in each neuropsychiatric domain on the PAI and the MINI between groups using Fisher's exact test (with Bonferroni correction for *post-hoc* pairwise testing). Clinical elevations on the PAI were operationalized as a scaled T-score of 70 or greater, based on conventional clinical criteria for the test (16), and clinical diagnoses were made based on MINI interview clinical diagnosis employing the test's diagnostic criteria (41).

For the MINI diagnoses, “current” and “previous” diagnoses were combined for each scale to prevent over-representation of a domain. Similarly, anxiety diagnoses other than Generalized Anxiety Disorder were combined into an “Anxiety—Other” scale, where Panic Disorder, Agoraphobia, Social Phobia, Obsessive Compulsive Disorder, and Posttraumatic Stress Disorder were collapsed into a single outcome variable. Mania and hypomania diagnoses were also collapsed into a single variable. An additional “Diagnoses—Other” outcome variable was created for the remaining disorders (but for which there were no cases diagnosed).

To describe the frequency of neuropsychological impairments, we computed the number of participants in the borderline/mild (−1.4 to −1.9 z-scores), moderate (−2 to −2.9 z-scores), and severe (−3 or more z-scores) ranges for each test, based on clinical convention (50). We then compared the proportions of mild, moderate, and severe neuropsychological impairments between groups using Fisher's exact test.

Objective 2: To examine the effects of concussion exposure, a higher concussion exposure group and a lower concussion exposure group was created by separating retired athletes according to number of self-reported concussions. Higher concussion exposure was operationally defined as 4 or more (*n* = 35) and lower concussion exposure was defined as 3 or fewer concussions (*n* = 26). Concussion number was determined from the player's recall of concussions as defined by McCrea et al. (51). Self-reported concussions are considered a moderately though not highly reliable index of concussion exposure (52). Therefore, we used a binary concussion exposure variable (high vs. low) rather than a continuous measure, based on the precedent of Guskiewicz et al. (12).

Using 1-way ANOVA, the higher and lower concussion exposure subgroups and the matched control group were compared on each of the demographic, neuropsychiatric, and neuropsychological variables (note that by chance, the subgroups remained demographically equivalent to one another and to the control group). We also compared the frequencies of PAI elevations, MINI clinical diagnoses, and neuropsychological impairments between the higher and lower concussion exposure subgroups and the control group using Fisher's exact test, as carried out between the full group of former players and control participants in Objective 1. For planned comparisons, we employed alpha levels as above.

Objective 3: Lastly, we investigated the relationship between executive and neuropsychiatric functioning as a function of concussion exposure in the former players, excluding controls. We first examined the contributions of concussion exposure and our experimental measures of executive functioning (SART commission errors and IIV) to neuropsychiatric scale scores from the PAI (*Depression, Mania*, and *Aggression*) using hierarchical linear regression. Separate regression analyses were run for each neuropsychiatric outcome, and predictor variables were entered in three steps in the following order: concussion exposure, then SART commission errors, and then SART IIV. In a follow-up set of analyses, we examined the mediating role of these novel executive functioning measures in the association between concussion exposure and neuropsychiatric functioning. Separate mediation analyses were performed for each neuropsychiatric variable previously included in the regression. Bootstrapping for mediation analysis with bias-corrected confidence estimates was performed using 5,000 bootstrap samples (53, 54).

## Results

We compared the demographic characteristics of the former professional football players to the control participants and found that the two groups were similar in age, educational attainment, and estimated premorbid IQ (see Table 1). After the former professional football players were divided into “higher concussion exposure” and “lower concussion exposure” subgroups, these subgroups did not differ significantly in age, education, or estimated premorbid IQ from one another or from control participants.

### Objective 1: Neuropsychiatric and Cognitive Characterization

Independent t-tests were applied to compare neuropsychiatric and cognitive functioning of the former professional football players and control participants. Consistent with our hypotheses, former players scored significantly higher than control participants on the PAI's *Depression, Mania*, and *Aggression* scales (after accounting for multiple comparisons, except where a 1-tailed test applied), with medium to large effect sizes (see Table 2). Former players and control participants did not differ significantly on any of the other neuropsychiatric variables. All three subscale variables for *Mania* (i.e., *Grandiosity, Irritability*, and *Activity*) and two of three subscales for *Aggression* (i.e., *Aggressive Attitude* and *Physical Aggression*, but not *Verbal Aggression*) differed significantly between the two groups after controlling for multiple comparisons. Thus, former professional football players with a history of multiple concussions reported significantly greater depression and mania symptoms, as well as aggression, than community control participants.

**Table 2:** Mean PAI clinical scale and sub-scale scores for former professional football players (*N* = 61) and matched control participants (*N* = 31).

	**All retired athletes *M* (*SD*)**	**Control participants *M* (*SD*)**	***T* (*df*), *p***	***d***
Depression^+^	49.3 (10.4)	44.7 (9.0)	2.09 (88), 0.040	0.47
Cognitive	47.6 (8.4)	45.9 (6.4)	0.99 (88), 0.324	0.23
Affective	50.0 (9.1)	46.6 (7.9)	1.75 (88), 0.042	0.40
Physiological	51.2 (10.9)	44.4 (9.8)	2.90 (88), 0.005	0.66
Anxiety	46.4 (8.1)	42.8 (8.7)	1.95 (88), 0.054	0.43
Anxiety-Related Disorders	46.0 (8.6)	42.5 (8.5)	1.81 (88), 0.074	0.41
Mania^+^	52.9 (10.2)	42.3 (8.2)	4.89 (88), <0.001	1.15
Activity Level	49.6 (9.0)	42.5 (9.2)	3.50 (88), 0.001	0.78
Grandiosity	56.8 (11.0)	46.3 (7.9)	4.62 (88), <0.001	1.10
Irritability	49.7 (9.9)	42.6 (8.5)	3.34 (88), 0.001	0.77
Paranoia	45.9 (8.0)	45.3 (8.8)	0.34 (88), 0.734	0.07
Borderline	47.4 (9.1)	43.3 (9.2)	2.01 (88), 0.047	0.45
Antisocial	50.1 (7.7)	47.6 (8.4)	1.41 (88), 0.163	0.31
Alcohol problems	53.5 (10.8)	48.9 (8.8)	2.01 (88), 0.047	0.47
Drug Problems	50.8 (9.1)	46.5 (5.2)	2.38 (88), 0.019	0.58
Aggression^+^	50.7 (11.0)	42.6 (6.0)	3.72 (88), <0.001	0.91
Aggressive Attitude	49.5 (12.1)	41.2 (6.2)	3.53 (88), 0.001	0.86
Verbal Aggression	51.4 (10.2)	46.1 (9.0)	2.39 (88), 0.019	0.55
Physical Aggression	50.7 (10.1)	44.3 (4.2)	3.29 (88), 0.001	0.83

*Mean T-scores presented for all clinical scales (minus Somatization and Schizophrenia) plus Aggression. Mean sub-scale scores only presented where full clinical scale scores differed between groups*.

+ *Denotes 1-tailed test applies. Means and standard deviations presented in the table are T-scores. CFL, Canadian Football League (indicates former professional football player group)*.

We compared the frequencies of PAI clinical elevations and MINI diagnoses for all former professional football players and control participants using Fisher's exact test (as operationalized in the Methods; see Supplementary Table 2). Our results showed that a significantly greater proportion of former players met criteria for one or more clinical diagnoses on the MINI compared to control participants (36% vs. 16%, respectively, *p* < 0.05). Furthermore, 11 of 61 former professional football players compared to one of 31 control participants met clinical criteria for a current or past manic/hypomanic episode on the MINI, a difference in proportions that was statistically significant (18% vs. 3%, respectively, *p* < 0.05). We then compared the former players' and control group's performance on each of the cognitive tests. The former professional football players made significantly more go/no-go commission errors on the SART than control participants, with a medium effect size, in partial support of our hypotheses (see Table 3). Our hypothesis that former players would show significantly worse scores on tests of learning and memory was not supported. These results suggest that former professional football players were less able to withhold a prepotent motor response on the experimental SART task (an index of inhibitory control) compared to control participants. Furthermore, the results showed that former players' learning and memory abilities were similar to those of the control participants.

**Table 3:** Mean raw scores on cognitive measures for former CFL players (*n* = 61) and control participants (*n* = 31)^a^.

	**All retired athletes *M* (*SD*)**	**Control participants *M* (*SD*)**	***T* (*df*), *p***	***d***
Go/no-go errors^+^	12.3 (5.7)	9.7 (4.9)	2.07 (88), 0.041	0.48
Go/no-go RT^+^	358.3 (92.4)	377.1 (80.9)	−0.95 (88), 0.346	−0.22
Go/no-go IIV^+^	28.2 (11.7)	24.9 (7.5)	1.43 (88), 0.156	0.34
RAVLT trials 1–5 total score^+^	45.1 (9.3)	46.9 (7.7)	−0.94 (90), 0.351	−0.21
RVDLT trials 1–5 total score^+^	39.4 (11.4)	39.9 (11.1)	−0.19 (90), 0.854	−0.04
SDMT-O total correct	61.2 (12.3)	63.3 (12.7)	−0.74 (90), 0.461	−0.16
Trails A total time (sec)	25.0 (7.5)	27.3 (11.1)	−1.19 (90), 0.238	−0.25
Trails B total time (sec)^+^	62.56 (23.7)	65.6 (25.4)	−0.56 (90), 0.579	−0.12
Spatial span forwards SS	11.4 (2.6)	11.3 (2.5)	0.07 (90), 0.947	0.02
Spatial span backwards SS	12.52 (2.8)	12.4 (2.8)	0.22 (90), 0.826	0.05
Digit span forwards %ile	63.5 (31.4)	52.1 (30.0)	1.67 (90), 0.098	0.37
Digit span backwards %ile	66.9 (27.8)	59.3 (24.9)	1.29 (90), 0.202	0.29

+ *Denotes 1-tailed test applies*.

a *SART scores for one control participant and one retired professional football player were invalid and therefore excluded. SART, Sustained Attention to Response Task; RT, Reaction Time; RAVLT, Rey Auditory Verbal Learning Test; RVDLT, Rey Visual Design Learning Test; SDMT-O, Symbol Digit Modalities Test-Oral; CFL, Canadian Football League (indicates former professional football player group)*.

We also compared the frequencies of cognitive impairments observed in the full retired professional football player group and control group. Fisher's exact test results indicated that there were no statistically significant differences in the frequencies of impairments between the two groups for any of the neuropsychological measures (results presented in Supplementary Table 3).

### Objective 2: Comparing Retired Athletes With Higher vs. Lower Concussion Exposure

**Figure 1:**
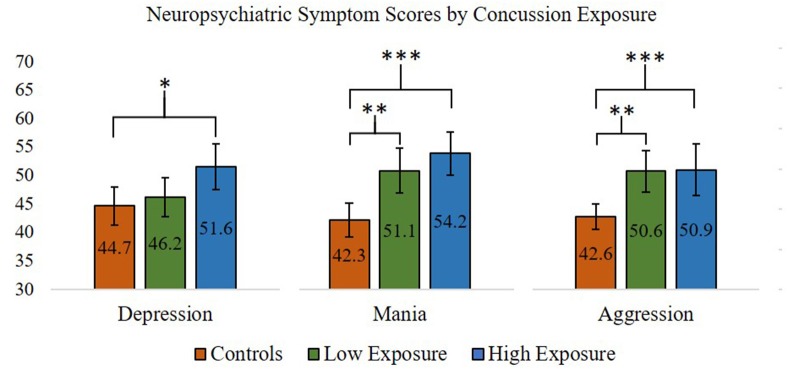
ANOVA and *post-hoc* test results comparing Personality Assessment Inventory Depression, Aggression, and Mania scale scores (presented in T-scores) between former professional football players with higher (>3) and lower (≤3) self-reported concussion exposure and control participants. ^*^p < 0.05; ^**^p < 0.01; ^***^p < 0.001. Error bars = 95% confidence intervals.

For the neuropsychiatric outcomes, we performed a series of one-way ANOVAs comparing concussion exposure subgroups and control participants for all PAI variables. Our results showed that *Depression* [*F*_(2, 87)_ = 4.63, *p* < 0.05], *Mania* [*F*_(2, 87)_ = 12.75, *p* < 0.001], and *Aggression* [*F*_(2, 87)_ = 6.83, *p* < 0.01] scores were significantly different between groups. According to Levene's statistic, the assumption of homogeneity of variances was violated for *Aggression*. Welch's *F* statistic confirmed that the difference between groups was still statistically significant [*F*_(2, 53)_ = 10.44, *p* < 0.001]. *Post-hoc* comparisons using the Tukey HSD test indicated that, for the former professional football players who reported 4 or more previous concussions, mean T-scores for the PAI's *Depression* (*M* = 51.71, *SD* = 11.13), *Mania* (*M* = 54.18, *SD* = 10.59), and *Aggression* (*M* = 50.71, *SD* = 12.51) scales were significantly greater than those observed for the control group (*Depression M* = 44.67, *SD* = 8.96; *Mania M* = 42.33, *SD* = 8.22; *Aggression M* = 42.63, *SD* = 5.95). Former players who reported 3 or fewer previous concussions had significantly higher scores on the PAI's *Mania* (*M* = 51.12, *SD* = 9.71) and *Aggression* (*M* = 50.62, *SD* = 9.02) scales, but not the *Depression* (*M* = 46.19, *SD* = 8.62) scale, compared to the control group. Thus, regardless of self-reported concussion exposure, the retired professional football players had significantly higher scores on the PAI's *Mania* and *Aggression* scales, which was not consistent with our hypotheses. However, only the retired players with four or more self-reported past concussions had significantly higher *Depression* scale scores compared to the control group, which was consistent with our hypotheses (see Figure 1 for planned comparisons results).

Regarding PAI elevations and MINI clinical diagnoses, a greater proportion of participants in the higher concussion exposure subgroup had one or more MINI clinical diagnoses compared to the control group (49% vs. 16%, respectively, *p* < 0.05). The proportion of participants with one or more MINI clinical diagnoses in the lower exposure subgroup did not differ significantly from the higher exposure subgroup or the control group. Furthermore, of the 11 former players who met criteria for a current or past manic/hypomanic episode, 10 were in the higher exposure subgroup, a proportion that was significantly greater than the lower concussion exposure subgroup (18% vs. 4%, respectively, *p* < 0.05) and the control group (18% vs. 3%, respectively, *p* < 0.05). No other differences in proportions of PAI elevations or MINI clinical diagnoses were found between the concussion exposure subgroups and control group.

**Figure 2:**
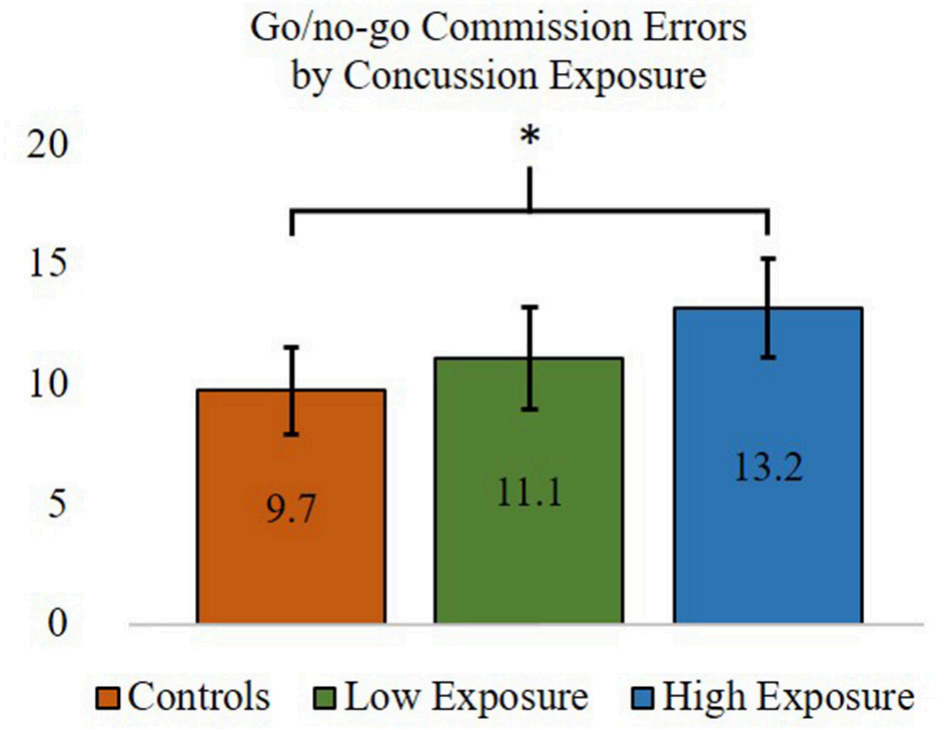
ANOVA and *post-hoc* test results comparing inhibitory control—commission errors (out of a possible 25) between former professional football players with higher (>3) and lower (≤3) self-reported concussion exposure and the control group. ^*^p < 0.05. Error bars = 95% confidence intervals.

Cognitive outcomes for the concussion exposure subgroups and control participants were performed using one-way ANOVAs. In partial support of our hypotheses, ANOVA results indicated that errors of commission, *F*_(2, 87)_ = 3.27, *p* < 0.05, but not IIV, *F*_(2, 87)_ = 3.04, *p* = 0.053 (approaching significance), on the SART was significantly different between groups. *Post-hoc* comparisons showed that retired players with higher self-reported concussion exposure made significantly more go/no-go commission errors (*M* = 13.18, *SD* = 5.95) than control participants (*M* = 9.73, *SD* = 4.88). Retired players with 3 or fewer reported previous concussions did not differ significantly from the higher concussion exposure subgroup or control group for go/no-go commission errors (lower exposure group *M* = 11.08, *SD* = 5.33). Frequencies of mild, moderate, and severe impairments for each of the cognitive variables were also compared between the higher and lower concussion exposure subgroups and the control group. Comparisons using Fisher's exact test indicated that there were no statistically significant differences in the frequency of impairments between groups for any of the cognitive variables. Thus, retired professional football players with four or more self-reported concussions were significantly worse at inhibiting a prepotent motor response (i.e., SART commission errors, inhibitory control) than control participants, whereas retired players with 3 or fewer self-reported concussions did not differ from those with 4 or more concussions or from control participants (see Figure 2). There were no differences between any of the groups on any other measure of cognitive functioning, including learning and memory, which did not support our hypotheses.

### Objective 3: Exploring the Relationship Between Concussion Exposure, Executive Functioning, and Neuropsychiatric Function

We performed hierarchical linear regression analyses to test models in which the former players' concussion exposure, SART commission errors, and SART IIV were used to predict their PAI *Depression, Aggression*, and *Mania* scale scores. The analyses yielded significant models in the prediction of PAI *Depression, F*_(3, 58)_ = 4.40, *p* < 0.01, and *Aggression, F*_(3, 58)_ = 3.14, *p* < 0.05, scores, and were marginal for the model predicting *Mania* scores, *F*_(3, 58)_ = 2.31, *p* = 0.086. Regression coefficients revealed that IIV was a significant predictor in the PAI *Depression* (R-squared change for the addition of IIV to the model was 0.067) and *Aggression* (R-square change for the addition of IIV was 0.121) models. No individual predictors were significant in the model for PAI *Mania* (results presented in Table 4).

**Table 4:** Hierarchical linear regression in the prediction of retired players' Depression, Mania, and Aggression scores on the Personality Assessment Inventory.

	**β**	**Δ*R*^**2**^**
**A. Regression predictors of PAI Depression**		
**Step 1**		
Concussion exposure	0.26^*^	0.066^*^
**Step 2**		
Concussion exposure	0.21	
SART commission errors	0.25	0.060
**Step 3**		
Concussion exposure	0.17	
SART commission errors	0.11	
SART IIV	0.30^*^	0.067^*^
Total *R*^2^: 0.194		
**B. Regression predictors of PAI Mania**		
**Step 1**		
Concussion exposure	0.15	0.022
**Step 2**		
Concussion exposure	0.11	
SART commission errors	0.21	0.042
**Step 3**		
Concussion exposure	0.07	
SART commission errors	0.09	
SART IIV	0.25	0.047
Total *R*^2^: 0.112		
**C. Regression predictors of PAI Aggression**		
**Step 1**		
Concussion exposure	0.01	0.000
**Step 2**		
Concussion exposure	−0.02	
SART commission errors	0.16	0.025
**Step 3**		
Concussion exposure	−0.08	
SART commission errors	−0.03	
SART IIV	0.40^**^	0.121^**^
Total *R*^2^: 0.146		

* p <0.05;

** *p <0.01. PAI, Personality Assessment Inventory; SART, Sustained Attention to Response Task*.

**Figure 3:**
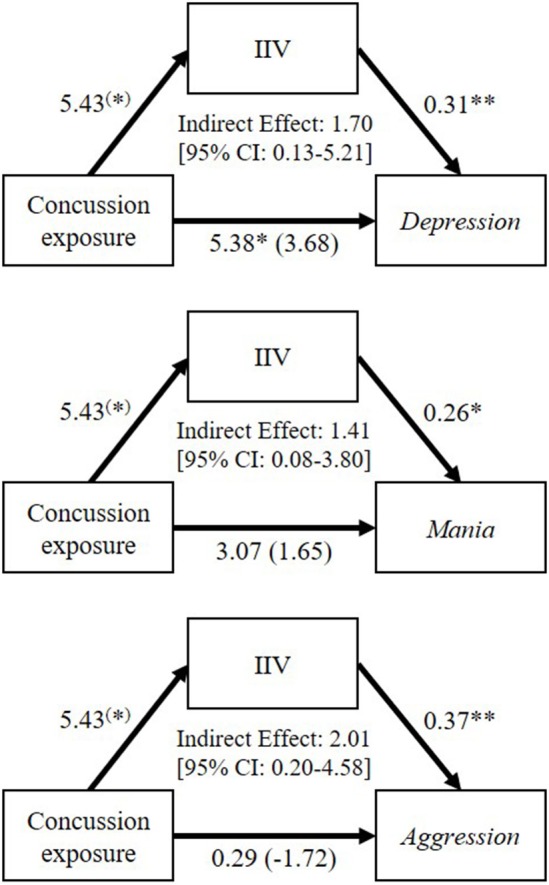
Mediation analyses to examine the mediating role of go/no-go reaction time intra-individual variability (IIV; inconsistency of responding) on the relationships between former professional football players' self-reported concussion exposure and their Depression, Mania, and Aggression scores on the Personality Assessment Inventory. Values accompanying each arrow represent unstandardized regression weights. The unstandardized regression coefficients between concussion exposure and each neuropsychiatric variable, controlling for IIV, are in parentheses. ^(*)^*p* < 0.10; ^*^p < 0.05; ^**^p < 0.01.

To address our final question, whether executive dysfunction was implicated in former players' neuropsychiatric symptoms, we examined whether IIV mediated the relationship between their concussion exposure and neuropsychiatric functioning. IIV was chosen as a mediating variable because it was the only significant predictor from the regression analysis. Results of the mediation analyses revealed a significant intervening effect of IIV in the relationship between concussion exposure and *Depression* (*B* = 1.70; CI = 0.13 to 5.21), *Mania* (*B* = 1.41; CI = 0.08 to 3.80), and *Aggression* (*B* = 2.01; CI = 0.20 to 4.58). The direct effect of concussion exposure on *Depression* (*B* = 3.68, *t*_(57)_ = 2.01, *p* = 0.161), *Mania* (*B* = 1.65, *t*_(57)_ = 0.62, *p* = 0.540), and *Aggression* (*B* = −1.72; *t*_(57)_ = −0.61, *p* = 0.544) was non-significant when controlling for IIV. Thus, concussion exposure had a significant indirect effect on *Depression, Mania*, and *Aggression* through IIV. These results are depicted in Figure 3.

## Discussion

This high-functioning group of retired professional Canadian football players showed disproportionate impairments to neuropsychiatric and executive functioning. The retired players showed significantly higher scores on the *Depression, Mania*, and *Aggression* scales of the PAI as compared to control participants. As well, more than double the percentage of retired players than control participants reached clinical threshold for a neuropsychiatric diagnosis on the PAI and on the MINI. Taken together, these results suggest a non-trivial neuropsychiatric burden in these high-functioning retired players that extends beyond the increased depressive symptoms reported in previous *in vivo* studies (11–13). Our findings are also compatible with the sporadic cases of *retrospectively* identified aggression and mania in post-mortem studies of retired football players (15).

Regarding the question of whether the findings above are attributable to having sustained multiple concussions or rather to pre-morbid characteristics of this unique cohort of individuals (i.e., retired CFL players), we examined the role of concussion exposure. We found a significant effect of exposure for the *Depression* clinical scale, but not the *Aggression* and *Mania* scale scores. However, on more fine-grained analyses, proportionately more former professional football players met threshold for a clinical diagnosis of mania/hypomania in the higher concussion exposure group than the lower exposure group. Moreover, unlike the low exposure group, the higher concussion exposure group had more clinical diagnoses on the MINI than control participants. Nonetheless, further research is needed to understand the role of concussion exposure (both number and severity) in neuropsychiatric functioning in this population, including research into the structural underpinnings of neuropsychiatric functioning given our prior pilot findings showing that higher aggression scores correlated negatively with orbitofrontal cortex thickness and uncinate fasciculus axial diffusivity (17). An alternative explanation for the current findings—that higher pre-morbid mania symptoms increase risk of sustaining more concussions—should be examined as well. In short, concussions appear to increase manifestation of certain neuropsychiatric characteristics. However, as elite football may select for traits associated with mania and aggression, some outcome variance may be explained by pre-morbid/cohort characteristics. Taken together, the above results suggest that people with a remote history of multiple concussions sustained in contact sports contend with non-trivial neuropsychiatric symptoms and should be considered for comprehensive neuropsychiatric assessment. Many efficacious treatments exist for neuropsychiatric dysfunction, both psychological (e.g., cognitive behavior therapy) and pharmacological (55, 56). Research is needed to evaluate the extent to which those with a remote history of multiple concussions sustained outside of contact sport may be suffering the same neuropsychiatric burden.

Turning to the cognitive characterization, the only measure that discriminated the full group of former professional football players from control participants was inhibitory control (i.e., commission errors on the SART). These results are partially consistent with a previous study in former professional football players, which found impairments to executive function, but not in the absence of other cognitive impairments (22). It was unexpected that no other cognitive measures, including learning and memory, discriminated former professional football players from control participants^1^ (18). The pattern of findings may reflect both the higher functioning of our sample compared to those in other studies (8, 18, 22) as well as the sensitivity of the SART task—and the importance of inhibitory control—in this population.

Although inconsistency of responding (calculated using IIV on the SART) did not discriminate the retired players from controls or the higher concussion exposure group from the lower exposure group (though marginally significant differences were observed), it *was* predictive of neuropsychiatric outcomes in the former players—in particular, PAI *Depression* and *Aggression* scores. We thus examined whether inconsistency of responding might mediate the relationship between concussion exposure and neuropsychiatric symptoms in the former players given that executive functioning has been implicated in neuropsychiatric dysfunction (31–33), and that in our pilot study of former professional football players, we found that executive function deficits and neuropsychiatric symptoms correlated with the same structural findings (17). A series of mediation analyses revealed that former players' concussion exposure had a significant indirect effect on their PAI *Depression, Aggression*, and *Mania* scores via inconsistency of responding. These results offer an interesting and novel hypothesis to be tested in future research: that impairment to one aspect of executive functioning (i.e., inconsistency of responding) renders individuals with a history of multiple concussions more vulnerable to the manifestation of neuropsychiatric symptoms including depression, mania, and aggression. In other words, in the absence of this deficit, these symptoms may not be manifested.

Our findings suggest that executive functioning should be assessed carefully, and with more than just traditional clinical measures; in this study, conventional measures were less sensitive than our experimental ones. Encouragingly, there are known treatments for executive dysfunction, including Goal Management Training (GMT) (57). GMT has been shown to improve executive functioning (measured using the same go/no-go task used in our study) in patients with frontal lobe brain damage (58). Notably, participants improved on inhibitory control (commission errors) from pre- to post-GMT, and benefits were maintained at 4-month follow-up (58). Participants also showed marginally significant improvements in inconsistency of responding (indexed by IIV) from baseline to follow-up. Another intervention, with the potential to improve both executive and neuropsychiatric functioning is mindfulness training (59–61), which has been shown to decrease inconsistency of responding (62, 63) and to improve response inhibition accuracy (63). Previous research has also demonstrated that mindfulness meditation may enhance emotion regulation through improvements in executive functioning (59), results consistent with our finding that self-reported concussion exposure was indirectly associated with neuropsychiatric symptoms via IIV.

Our study has limitations that affect the generalizability of the current findings. The use of self-reported concussion history is problematic because recollection of concussion is considered only moderately reliable (52). We sought to address this limitation with our use of a binary concussion history variable as opposed to a continuous variable, a method for estimating concussion exposure employed in previous studies (12, 20). The current study may also be limited by self-selection biases. For example, participants in this study may represent former CFL players who were experiencing neuropsychiatric and cognitive concerns and therefore sought out a research study that would help to address them. It should be noted, however, that our sample comprised retired players with a history of concussion who were high-functioning (based on self-report of current occupational and social functioning). Most of the players reported that they entered the study to support their fellow players. Another potential limitation was our use of community control participants, as opposed to professional athlete control participants without a history of concussion. To help address this issue, we conducted subgroup comparisons within the former professional football player group to differentiate former players as a function of concussion exposure. Comparing individuals within the same population was done in an effort to distinguish cognitive and neuropsychiatric findings attributable to concussion exposure vs. cohort characteristics (64). Finally, in the current cohort study, we cannot ascertain whether the selective impairments observed represent the residual effects of original concussions or a *de novo* disorder, perhaps associated with the aging process.

In sum, we have presented a comprehensive neuropsychiatric and cognitive profile of high-functioning former professional football players and a disproportionate, but treatable, burden of neuropsychiatric and executive function deficits (the latter revealed on an experimental measure of executive function, the SART). Our findings also revealed a novel potential treatment target for remediating neuropsychiatric deficits—inconsistency of responding—that warrants further research. Future research should also examine the extent to which these findings generalize to those with a remote history of multiple concussions sustained *outside* the context of contact sport.

From a neurorehabilitative point of view, people with a remote history of multiple concussions, particularly when sustained in the context of contact sport, should be considered for comprehensive neuropsychiatric assessment (and treatment) and for careful evaluation of executive dysfunction, with an emphasis on both inhibitory control and inconsistency of responding (i.e., IIV).

## Data Availability

The datasets generated for this study are available upon request to the corresponding author.

## Ethics Statement

This study was approved by the University Health Network Research Ethics Board, Toronto, ON, Canada.

## Consent Statement

All participants of this study provided their informed consent in person prior to the face-to-face study assessment and written informed consent.

## Author Contributions

RG, AT, BC, and BV contributed conception and design of the study. BC and AT organized the database. AT and BV performed the statistical analyses. RG and AT wrote the first draft of the manuscript. BV wrote sections of the manuscript. MT, CT, KD, DM, RG, and RW were involved in the conceptualization and data collection for the larger study, of which the current study is a part. All authors contributed to manuscript reviewing and editing.

### Conflict of Interest Statement

The authors declare that the research was conducted in the absence of any commercial or financial relationships that could be construed as a potential conflict of interest.

## Footnotes

^1^Given this deviation from past findings, we attempted to obtain a more sensitive index of memory impairment by individually bench-marking memory function against estimated pre-morbid IQ (i.e., an IQ-MQ split). However, again, there was no significant difference between the groups.
