# Supplementary material for: Comprehensive Neuropsychiatric and Cognitive Characterization of Former Professional Football Players: Implications for Neurorehabilitation
Source: Front Neurol. 2019 Aug 7;10:712. doi: 10.3389/fneur.2019.00712 (PMC6696977; doi:10.3389/fneur.2019.00712)
Supplement: Supplementary file 1 [file Data_Sheet_1.docx]

Supplementary Material

# Supplementary Tables

## Supplementary Table 1. *Neuropsychological assessment battery by domain of cognitive functioning.* Cognitive domain, test name plus respective dependent variable used in analyses, plus brief test description provided.

| Domain | Test (dependent variable) | Test description |
| --- | --- | --- |
| Attention and speed of processing | Trail Making Test—A  (time) (1) | Timed psychomotor test of simple visual attention |
|  | Digit Span—Forwards  (total correct) (1) | Untimed auditory test of auditory-verbal attention |
|  | Spatial Span—Forwards  (total correct) (1) | Untimed manual motor test of visual attention |
|  | Symbol Digit Modalities Test—Oral (total correct items) (2) | Timed oral test of processing speed |
| Visual spatial and verbal memory | Rey Auditory Verbal Learning Test (2) (total items recalled) | Untimed, non-motor test of auditory verbal learning, recall, and recognition for unorganized verbal information |
|  | Rey Visual Design Learning Test (2) (total items recalled) | Untimed, manual motor test of visual learning, recall, and recognition for unorganized visual information |
| Executive functioning  Traditional measures  Experimental measures | Trail Making Test—B  (time) (1) | Timed motor test of mental flexibility and set shifting |
|  | Digit Span—Backwards  (total correct) (1) | Untimed test of auditory working memory |
|  | Spatial Span—Backwards (total correct) (1) | Untimed sequence tapping test of visual spatial working memory |
|  | Sustained Attention to Response Task (3) | Timed, go-no/go, manual motor test of (i) inhibitory control and (ii) response time intra-individual variability |
| Estimated pre-morbid intellectual function | Wechsler Test of Adult Reading (4) | Untimed test of oral word pronunciation; validated for people with a history of TBI |
| Current intellectual functioning | Shipley Institutes of Living Scale (5) | Untimed measure of intellectual ability |

*Note.* For a minority of participants who were not administered the Wechsler Test of Adult Reading, the Shipley Institute of Living Scale was administered as a measure of IQ. TBI, traumatic brain injury.

## Supplementary Table 2. *Frequency of elevations on PAI clinical scales and on the MINI for retired athlete full group, exposure sub-groups, and age- and education-matched control participants.*

|  | All retired athletes  (n=61) | Low  concussion exposure  (n=26) | High  concussion exposure  (n=35) | Control participants (n=31) | Fisher’s Exact Test  *P* (All retired athletes vs. Control participants) | Fisher’s Exact Test  *P* (Low exposure vs. High exposure vs. Control participants) |
| --- | --- | --- | --- | --- | --- | --- |
| **Total number (percentage) of subjects with clinical elevations on one or more scales**^+^ | 15 (25%) | 5 (19%) | 10 (29%) | 3 (10%) | .161 | .115 |
| **Number (percentage) of clinical elevations by scale** |  |  |  |  |  |  |
| *Anxiety* | 1 (2%) | 0 (0%) | 1 (3%) | 0 (0%) | >.999 | >.999 |
| *Anxiety-Related Disorder* | 0 (0%) | 0 (0%) | 0 (0%) | 0 (0%) | N/A | N/A |
| *Depression*^+^ | 3 (0%) | 0 (0%) | 3 (9%) | 2 (7%) | >.999 | .374 |
| *Mania*^+^ | 5 (8%) | 1 (4%) | 4 (12%) | 0 (0%) | .165 | .123 |
| *Paranoia* | 0 (0%) | 0 (0%) | 0 (0%) | 0 (0%) | N/A | N/A |
| *Borderline* | 1 (2%) | 0 (0%) | 1 (3%) | 1 (3%) | >.999 | >.999 |
| *Antisocial* | 1 (2%) | 0 (0%) | 1 (3%) | 1 (3%) | >.999 | >.999 |
| *Alcohol Problems* | 5 (8%) | 1 (4%) | 4 (12%) | 1 (3%) | .659 | .371 |
| *Drug problems* | 4 (7%) | 2 (8%) | 2 (6%) | 0 (0%) | .297 | .453 |
| *Aggression*^+^ | 5 (8%) | 0 (0%) | 5 (14%) | 0 (0%) | .165 | .123 |
|  | All retired athletes  (n=61) | Low  concussion exposure  (n=26) | High  concussion exposure  (n=35) | Control participants (n=31) | Fisher’s Exact Test  *P* (All retired athletes vs. Control participants) | Fisher’s Exact Test  *P* (Low exposure vs. High exposure vs. Control participants) |
| **Total number (percentage) of subjects who meet criteria for *any* MINI diagnosis**^+^ | 22 (36%)^a^ | 5 (19%) | 17 (49%)^b^ | 5 (16%)^a,b^ | .038 | .004 |
| **Number (percentage) of clinical diagnoses** |  |  |  |  |  |  |
| *Depressive episode – current or previous*^+^ | 4 (7%) | 0 (0%) | 4 (11%) | 1 (3%) | .450 | .109 |
| *Dysthymia – current* | 3 (5%) | 0 (0%) | 3 (9%) | 0 (0%) | .548 | .108 |
| *Suicidality – current* | 1 (2%) | 0 (0%) | 1 (3%) | 1 (3%) | >.999 | >.999 |
| *Manic/hypomanic episode – current or previous*^+^ | 11 (18%)^a^ | 1 (4%)^b^ | 10 (30%)^b,c^ | 1 (3%)^a,c^ | .038 | .001 |
| *Generalized anxiety disorder* | 2 (3%) | 0 (0%) | 2 (6%) | 2 (7%) | .601 | .551 |
| *Anxiety disorder (other)* | 4 (7%) | 1 (4%) | 3 (9%) | 0 (0%) | .296 | .310 |
| *Alcohol dependence/abuse* | 11 (18%) | 3 (12%) | 8 (23%) | 2 (7%) | .206 | .157 |
| *Substance dependence/abuse* | 5 (8%) | 2 (8%) | 3 (9%) | 0 (0%) | .163 | .260 |
| *Diagnoses – Other (Psychotic, eating disorder)* | 0 (0%) | 0 (0%) | 0 (0%) | 0 (0%) | N/A | N/A |

*Note. “*Clinical elevation” operationally defined as 20 T-scores above the normed mean (6). PAI, Personality Assessment Inventory; MINI, Mini International Neuropsychiatric Inventory. ^+^Denotes 1-tailed test applies. Each superscript letter denotes categories whose column proportions differed significantly from each other at the *p* < .05 level.

## Supplementary Table 3. *Frequencies of borderline/mild, moderate, and severe impairments on neuropsychological tests for the full group of retired players, the concussion exposure sub-groups and control participants.*

|  |  | All retired athletes (n=61) | Low concussion exposure (N=26) | High concussion exposure (N=35) | Control group (n=31) | Fisher’s Exact Test  (All retired athletes vs. Control participants) | Fisher’s Exact Test  (Low exposure vs. High exposure vs. Control participants) |
| --- | --- | --- | --- | --- | --- | --- | --- |
| **Number (percentage) of mild, moderate, and severe impairments** |  |  |  |  |  |  |  |
| Go/no-go errors^+^ |  |  |  |  |  |  |  |
| Mild |  | 7 (12%) | 4 (15%) | 3 (9%) | 2 (7%) | .068 | .129 |
| Moderate |  | 6 (10%) | 2 (8%) | 4 (12%) | 0 (0%) |  |  |
| Severe |  | 0 (0%) | 0 (0%) | 0 (0%) | 0 (0%) |  |  |
| Go/no-go RT^+^ |  |  |  |  |  |  |  |
| Mild |  | 5 (8%) | 0 (0%) | 5 (14%) | 0 (0%) | .109 | .006 |
| Moderate |  | 0 (0%) | 0 (0%) | 0 (0%) | 0 (0%) |  |  |
| Severe |  | 1 (2%) | 0 (0%) | 1 (3%) | 0 (0%) |  |  |
| Go/no-go IIV^+^ |  |  |  |  |  |  |  |
| Mild |  | 4 (7%) | 1 (4%) | 3 (9%) | 0 (0%) | .175 | .129 |
| Moderate |  | 3 (5%) | 0 (0%) | 3 (9%) | 0 (0%) |  |  |
| Severe |  | 4 (7%) | 0 (0%) | 4 (12%) | 1 (3%) |  |  |
|  |  | All retired athletes (n=61) | Low concussion exposure (N=26) | High concussion exposure (N=35) | Control group (n=31) | Fisher’s Exact Test  (All retired athletes vs. Control participants) | Fisher’s Exact Test  (Low exposure vs. High exposure vs. Control participants) |
| RAVLT^+^ |  |  |  |  |  |  |  |
| Mild |  | 2 (3%) | 1 (4%) | 1 (3%) | 0 (0%) | .350 | .221 |
| Moderate |  | 1 (2%) | 1 (4%) | 0 (0%) | 0 (0%) |  |  |
| Severe |  | 0 (0%) | 0 (0%) | 0 (0%) | 0 (0%) |  |  |
| RVDLT^+^ |  |  |  |  |  |  |  |
| Mild |  | 7 (16%) | 4 (15%) | 3 (9%) | 2 (7%) | .271 | .234 |
| Moderate |  | 0 (0%) | 0 (0%) | 0 (0%) | 1 (3%) |  |  |
| Severe |  | 1 (2%) | 1 (4%) | 0 (0%) | 0 (0%) |  |  |
| SDMT-O |  |  |  |  |  |  |  |
| Mild |  | 0 (0%) | 0 (0%) | 0 (0%) | 0 (0%) | N/A | N/A |
| Moderate |  | 0 (0%) | 0 (0%) | 0 (0%) | 0 (0%) |  |  |
| Severe |  | 0 (0%) | 0 (0%) | 0 (0%) | 0 (0%) |  |  |
| Trail Making Test—A |  |  |  |  |  |  |  |
| Mild |  | 1 (2%) | 0 (0%) | 1 (3%) | 3 (10%) | .052 | .263 |
| Moderate |  | 0 (0%) | 0 (0%) | 0 (0%) | 1 (3%) |  |  |
| Severe |  | 1 (2%) | 1 (4%) | 0 (0%) | 1 (3%) |  |  |
|  |  | All retired athletes (n=61) | Low concussion exposure (N=26) | High concussion exposure (N=35) | Control group (n=31) | Fisher’s Exact Test  (All retired athletes vs. Control participants) | Fisher’s Exact Test  (Low exposure vs. High exposure vs. Control participants) |
| Trail Making Test—B |  |  |  |  |  |  |  |
| Mild |  | 1 (2%) | 0 (0%) | 1 (3%) | 0 (0%) | .563 | .858 |
| Moderate |  | 0 (0%) | 0 (0%) | 0 (0%) | 0 (0%) |  |  |
| Severe |  | 0 (0%) | 0 (0%) | 0 (0%) | 1 (3%) |  |  |
| Spatial Span Forwards |  |  |  |  |  |  |  |
| Mild |  | 1 (2%) | 1 (4%) | 0 (0%) | 0 (0%) | >.999 | .283 |
| Moderate |  | 0 (0%) | 0 (0%) | 0 (0%) | 0 (0%) |  |  |
| Severe |  | 0 (0%) | 0 (0%) | 0 (0%) | 0 (0%) |  |  |
| Spatial Span Backwards |  |  |  |  |  |  |  |
| Mild |  | 0 (0%) | 0 (0%) | 0 (0%) | 0 (0%) | N/A | N/A |
| Moderate |  | 0 (0%) | 0 (0%) | 0 (0%) | 0 (0%) |  |  |
| Severe |  | 0 (0%) | 0 (0%) | 0 (0%) | 0 (0%) |  |  |
| Digit Span Forwards |  |  |  |  |  |  |  |
| Mild |  | 1 (2%) | 0 (0%) | 1 (3%) | 3 (10%) | .120 | .260 |
| Moderate |  | 0 (0%) | 0 (0%) | 0 (0%) | 0 (0%) |  |  |
| Severe |  | 0 (0%) | 0 (0%) | 0 (0%) | 0 (0%) |  |  |
|  |  | All retired athletes (n=61) | Low concussion exposure (N=26) | High concussion exposure (N=35) | Control group (n=31) | Fisher’s Exact Test  (All retired athletes vs. Control participants) | Fisher’s Exact Test  (Low exposure vs. High exposure vs. Control participants) |
| Digit Span Backwards |  |  |  |  |  |  |  |
| Mild |  | 2 (4%) | 1 (4%) | 1 (3%) | 0 (0%) | .540 | .746 |
| Moderate |  | 0 (0%) | 0 (0%) | 0 (0%) | 0 (0%) |  |  |
| Severe |  | 0 (0%) | 0 (0%) | 0 (0%) | 0 (0%) |  |  |

*Note.* RT, Reaction Time; IIV, intra-individual variability; RAVLT, Rey Auditory Verbal Learning Test; RVDLT, Rey Visual Design Learning Test; SDMT-O, Symbol Digit Modalities Test-Oral. Impairment for go/no-go reaction time was operationalized as faster speed (7). ^+^Denotes 1-tailed test applies.

# References

1. Wechsler D. WAIS-3., WMS-3: Wechsler Adult Intelligence Scale, Wechsler Memory Scale: Technical Manual: psychological corporation; 1997.

2. Lezak MD. Neuropsychological assessment. USA: Oxford University Press; 2004.

3. Robertson IH, Manly T, Andrade J, Baddeley BT, Yiend J. Oops!': performance correlates of everyday attentional failures in traumatic brain injured and normal subjects. Neuropsychologia. 1997;35(6):747-58.

4. Wechsler D. Wechsler Test of Adult Reading: WTAR: Psychological Corporation; 2001.

5. Shipley WC. A self-administering scale for measuring intellectual impairment and deterioration. The Journal of Psychology. 1940;9(2):371-7.

6. Morey LC. Personality assessment inventory (PAI). Lutz, FL: PAR; 1991.

7. Carter L, Russell PN, Helton WS. Target predictability, sustained attention, and response inhibition. Brain and cognition. 2013;82(1):35-42.
